# Omalizumab ensures compatibility to bee venom immunotherapy (VIT) after VIT-induced anaphylaxis in a patient with systemic mastocytosis 

**DOI:** 10.5414/ALX02196E

**Published:** 2021-03-11

**Authors:** Askin Gülsen, Franziska Ruëff, Uta Jappe

**Affiliations:** 1 Interdisciplinary Allergy Outpatient Clinic, Department of Pneumology, University of Luebeck,; 2 Department of Dermatology and Allergology, Klinikum der Ludwig-Maximilians-Universität, Munich, and; 3 Division of Clinical and Molecular Allergology, Research Center Borstel, Leibniz Lung Center, Airway Research Center North (ARCN), German Center for Lung Research (DZL), Borstel, Germany

**Keywords:** bee venom allergy, mastocytosis, anaphylaxis, omalizumab, tolerance

## Abstract

Background: Systemic reactions and anaphylaxis due to Hymenoptera venoms occur in up to 7.5% of the European population. Fatal sting reactions are very rare. Serum tryptase levels should be measured in all patients with a history of severe reactions in order to detect mastocytosis and to determine the risk of severe reactions to venom immunotherapy (VIT). The risk to experience severe or even fatal anaphylaxis due to insect stings is quite high in patients with mastocytosis. Therefore, lifelong VIT is recommended in these highly threatened patients. Multicenter studies involving a large population report that up to 20% of patients undergoing VIT have intolerance and systemic reactions to immunotherapy. Some of these side effects occur repeatedly and cannot be managed by standard treatment. A pre-treatment with the anti-IgE antibody omalizumab was useful in many cases. However, omalizumab is not approved for the indication anaphylaxis. Therefore, there is still no defined protocol for omalizumab pre-treatment, and the optimal duration, dosage as well as long-time benefits are still unclear. Case report: We present a 60-year-old female patient with mastocytosis who developed a severe anaphylactic reaction during initiation of bee VIT. Serum tryptase was elevated, and a KIT mutation D816V was subsequently confirmed. Component-resolved diagnostic tests revealed specific IgE antibodies to recombinant Api m 1 only. The patient was treated with 150 mg omalizumab, administered subcutaneously 5 weeks, 3 weeks, and 1 week prior to re-start of immunotherapy and for 2 months in parallel to VIT. Updosing was done by a 7-day rush schedule. During this period, no anaphylactic reaction developed, and the bee VIT was well tolerated with up to 200 µg bee venom. The patient is currently in the 3^rd^ year of treatment and tolerates the treatment very well. Conclusion: Omalizumab may be used as a premedication in patients with mastocytosis who do not tolerate VIT. Although there is no consensus on the treatment protocol, treatment for 2 – 6 months is considered adequate. The long-term benefits of such treatment require further research.

## Introduction 

Systemic anaphylactic reactions due to Hymenoptera stings occur in 3.3% of the population of the United States, and 0.3 – 7.5% of the European population. The mortality rate is very low between 0.03 and 0.48 per 1,000,000 people [1, 2]. In particular, beekeepers are very much at risk to experience systemic reactions with a prevalence between 14 and 43% [1]. According to guidelines, venom immunotherapy (VIT) is indicated for patients with anaphylactic reactions and positive skin tests or evidence of venom-specific immunoglobulin E (IgE) [3]. Serum tryptase levels should be measured in all adult patients with a history of a severe reaction in order to diagnose possible mastocytosis and thereby to identify patients at particular risk [3]. Age, individual risk factors, and loss of quality of life are another important aspects in making a decision of VIT. 

Multicenter studies involving larger numbers of patients report that up to 20% of patients undergoing VIT have systemic reactions to immunotherapy [4]. The majority of these reactions are mild and occur only once or twice. Some of these systemic reactions, however, may lead to severe systemic anaphylactic reactions, requiring emergency interventions and eventually resulting in an early discontinuation of VIT. Omalizumab treatment (OT) has been used to improve tolerance in patients with anaphylactic reactions to VIT [5, 6, 7]. 

There are case reports in the literature about the benefits of OT in patients with mastocytosis and anaphylactic reactions to VIT [8, 9]. Some authors, however, could not gain positive experiences by pre-treatment with omalizumab. Although the application of omalizumab in the context of allergen-specific immunotherapy is already recommended in the guideline [4], it is still an off-label use. Due to a lack of prospective studies there is no validated protocol for OT in combination with VIT, the optimal duration, dosage as well as long-time benefits are unclear. 

Here we present a female patient with mastocytosis who had developed an anaphylactic reaction to initial bee VIT so that VIT had been discontinued and who achieved successful treatment tolerance after premedication with omalizumab. 

## Case report 

A 60-year-old woman was admitted to our Allergy Outpatient Clinic due to bee venom allergy and VIT intolerance already at low doses. The patient had experienced circulatory collapse and unconsciousness a few minutes after a bee sting in 2006 and again in 2016 requiring adrenaline for treatment. The patient had received bee stings while she had assisted her husband in beekeeping. Her husband had an apiary in an approximately 5-minutes walking distance from the place of residence. The patient was immediately treated and recovered without sequelae in both situations. Her past medical history revealed hypertension, which was under control by treatment with candesartan. 

In December 2016, she presented to an external allergy outpatient clinic. The allergy diagnostic tests revealed a sensitization to bee venom (specific IgE to bee venom extract: 2.28 kU/L (CAP Class 2); to rApi m 1: 2.47 kU/L (CAP Class 2)). Intracutaneous test for bee venom showed positive results from a dosage of 0.001 μg/mL (1 : 100,000) onwards, and the patients was advised to start an inpatient allergen-specific immunotherapy. VIT (Reless-Bee venom, ALK-Abelló Arzneimittel GmbH, Hamburg, Germany) was initiated according to the rush protocol. The patient tolerated the first-day doses very well. In the following days, the concentration and the applied injection volume reached 0.7 mL (10 μg/mL). After the last application, within a few minutes, there was initially a pronounced malaise with nausea, then the patient collapsed. She was then admitted to and treated at the intensive care unit, where her symptoms rapidly improved. VIT was discontinued due to the severe anaphylaxis. She was discharged with an emergency kit. 

Laboratory investigations had revealed an elevated serum tryptase (17.9 µg/L; reference range < 11.4 µg/L). Subsequently, a bone marrow biopsy was performed to investigate for mastocytosis, and as a result, indolent systemic mastocytosis was diagnosed. The diagnostic criteria were fulfilled: multifocal mast cell aggregates (with > 15 mast cells/aggregate, major criterion), partly spindle-shaped mast cells and an aberrant expression of CD25 on the mast cells (minor criterion). Molecular genetic testing had confirmed systemic mastocytosis (KIT mutation D816V). No skin involvement was observed in the patient. 

In August 2017, the patient presented to our allergy clinic to initiate VIT once again. Allergy diagnostic tests had revealed a normal total serum IgE concentration (11.4 kU/L; reference range < 100 kU/L), an elevated serum tryptase (24.1 µg/L), and elevated bee venom-specific IgE concentrations (to bee venom extract: 1.18 kU/L (CAP class 2); to rApi m 1: 1.21 kU/L (CAP class 2)). Specific IgE to other bee venom compounds (to rApi m2, rApi m 3, rApi m 5, and rApi m 10), as well as to wasp venom (wasp venom extract, rVes v 1, and rVes v 5) were not detectable (Table 1). A skin prick test revealed a positive reaction to bee venom at a concentration of 10.0 µg/mL. 

Because of the systemic mastocytosis, a lifelong bee VIT with 200 µg was indicated. However, since the patient had severely reacted during the first attempt to initiate bee VIT, we decided to perform a pretreatment with omalizumab (Table 2). The patient was treated with 150 mg omalizumab administered subcutaneously 5 weeks, 3 weeks, and 1 week prior to re-start of immunotherapy. VIT initiation was done by a rush protocol over 7 days up to 200 µg of venom. During this period, no anaphylactic reaction occurred, and the treatment was well tolerated. At months 1 and 2, omalizumab was administered before VIT, and since May 2018, only bee VIT injections (monthly 200 µg) were continued without any systemic reactions. At follow-up, serum tryptase concentrations were continuously elevated without significant alterations. The patient is currently close to the 4^th^ year of treatment and tolerates the treatment very well. We repeated the in vitro molecular allergy diagnostic parameters during treatment, and the data are presented in Table 1. 

## Discussion 

This case report demonstrates i.) that severe reactions to bee stings associated with mastocytosis do not necessarily present with allergen-specific IgE in high concentrations, and ii.) omalizumab was a useful medication able to induce tolerance to VIT in a patient with mastocytosis who had developed severe anaphylaxis to VIT before. The treatment protocol (duration and dosage) and the decision as to when the omalizumab treatment is to be discontinued is presently not supported by prospective studies. In a retrospective case series on this subject, it was recommended that OT should be administered according to the patient’s total IgE and body weight 5 weeks, 3 weeks, and 1 week before VIT initiation and applied for 3 – 6 months [10]. We acted in accordance with this protocol and terminated OT after 3 months. In addition, we chose the slower 7-day rush protocol, which we considered to be safer. 

Not in all treated patients with mastocytosis, 3 – 6 months duration and 150 mg of OT had been sufficient to obtain long lasting tolerance of bee VIT after OT discontinuation. Notably, in a case report published by Kontou-Fili et al. [9], 300 mg of omalizumab (twice the recommended dose) was administered 40 – 60 minutes before VIT to a patient with mastocytosis. At the 9^th^ month of treatment, the dose of omalizumab was reduced to 150 mg due to the good tolerance of VIT. After another 3 months of 150 mg OT and 100 µg of bee venom application, the patient developed mild tachycardia, facial flushing, and a local injection site reaction; therefore, OT was increased back to 300 mg. This treatment continued for a total of 26 months during which the patient remained completely free of any allergic reactions and adverse events. Whether patients develop permanent tolerance warrants future investigations. 

Our patient tolerated bee VIT well after discontinuation of the omalizumab treatment. We consider it essential to increase the maintenance dose of the venom while the patient is protected by the OT in order to achieve venom tolerance. 

A successful immunomodulatory effect was achieved in patients who did not develop systemic anaphylactic reactions after discontinuation of omalizumab. Most probably, some patients need a venom concentration higher than the standard dose of 100 µg and develop allergic side effects where the dose is not adapted to their individual reactivity level. Particularly for bee venom allergic patients, the standard dose often is not sufficient. OT can be understood as passive immunotherapy but does not replace active immunotherapy. If VIT is not safe and effective with a standard dose, a higher dose will provide better tolerance of treatment and later stings. Only where a venom tolerance has been achieved, omalizumab treatment can be safely stopped. 

However, it is not known exactly, how OT should be applied to patients who react to VIT after dose reduction or discontinuation of omalizumab. Prospective studies on OT dosage and treatment intervals for its use in combination with VIT are needed. Only if solid data exist and OT is approved for the indication anaphylaxis by the authorities, its status of “off-label-use” can be discontinued, and there will be no further hindrance regarding health insurance reimbursement. 

Anti-IgE antibodies bind to specific IgE antibodies, neutralize their effect and lead to a reduced risk of anaphylaxis due to VIT and natural exposure to allergens. 

Initial studies on the association between component-specific IgE reduction and treatment efficacy so far are interesting [11]. However, in this respect, more investigations have still to be performed. 

In our patient, concentrations of IgE specific for the single allergen components as well as bee venom extract were reduced, and no additional sensitization occurred. However, the significance of IgE to single venom allergens regarding treatment monitoring and efficacy is still under investigation. Even if the IgG4 concentration to some components increased [11, 12], this is not a reliable parameter for protection. Another important issue is the predictive value of molecular allergy diagnostics for safety and efficacy of VIT. Very low levels (< 1 ISU or < 2 kU_A_/L) of IgE to Api m 1 and/or Ves v 5 can be seen in patients with severe reactions [13]. In addition, a decrease in specific IgE (Api m 1 and Api m 4) concentrations is reported 1 year after venom immunotherapy [14]. Therefore, it does not seem easy to predict the next reaction with venom-specific IgE concentrations at present. 

## Conclusion 

Different clinical pathologies such as systemic mastocytosis should be sought in patients who develop anaphylaxis against treatment, as in our patient. Omalizumab may be used as a premedication in patients with mastocytosis who do not tolerate VIT. Although there is no consensus on the treatment protocol, treatment for 3 – 6 months is considered adequate if VIT is done with a higher maintenance dose. The long-term benefits of such treatment require further research. 

## Funding 

Funding of the Federal Ministry for Science and Education (BMBF), German Center for Lung Research, is gratefully acknowledged. 

## Conflict of interest 

The authors have stated explicitly that there are no conflicts of interest in connection with this article. 

**Table 1.** Molecular allergy diagnostics.

Parameter	Extern 09-2016	08-2017	09-2019	06-2020
Tryptase (µg/L)	17.9	24.1	22.4	27.5
Total IgE (kU/L)	4.1	11.4	10.9	10.6
	IgE to extracts and allergen components			
Bee venom-extract	2.28	1.18	0.37	0.31
rApi m 1 (kU/L)	2.47	1.21	0.33	0.27
rApi m 2 (kU/L)	–	< 0.01	< 0.01	< 0.01
rApi m 3 (kU/L)	–	< 0.01	< 0.01	< 0.01
rApi m 5 (kU/L)	–	< 0.01	< 0.01	< 0.01
rApi m 10 (kU/L)	–	< 0.01	< 0.01	< 0.01
Bee venom IgG (mg/L)	–	2.8 (R: < 10.0)	13.1 (R: < 10.0)	16.6 (R: < 10.0)
Bee venom IgG4 (mg/L)	–	0.43 (R < 0.1)	6.97 (R < 0.1)	9.43 (R < 0.1)
Wasp venom extract	< 0.01	< 0.01	< 0.01	< 0.01
rVes v 1 (kU/L)	–	< 0.01	< 0.01	< 0.01
rVes v 5 (kU/L)	< 0.001	< 0.01	< 0.01	< 0.01
Wasp venom IgG (mg/L)	–	4.7 (R: < 10.0)	4.3 (R: < 10.0)	5.1 (R: < 10.0)
Wasp venom IgG4 (mg/L)	–	0.24 (R < 0.1)	0.19 (R < 0.1)	0.16 (R < 0.1)
CCD MUXF3 (kU/L)	–	< 0.01	< 0.01	–

Allergen component data was given as kU/L. Ig = immunoglobulin; R = reference; CCD = cross-reactive carbohydrate determinants.

**Table 2.** Synopsis of the treatment protocol.

Date	Therapy	Symptoms after last injection
5 weeks before VIT 23.01.2018	Omalizumab 150 mg	None
3 weeks before VIT 06.02.2018	Omalizumab 150 mg	None
1 week before VIT 20.02.2018	Omalizumab 150 mg	None
Week 0 27.02.2018	Rush-VIT protocol (Scheme up to 200 µg, 7-day protocol)	None
Week 4 19.03.2018	Omalizumab 150 mg (3 days before VIT)	None
Week 4 22.03.2018	VIT 100 µg (left) + 100 µg (right)	None
Week 8 24.04.2018	Omalizumab 150 mg + VIT 200 µg	None
Monthly Until today	VIT 200 µg (life-long)	None

VIT = venom immunotherapy.
